# Root-Colonizing Endophytic Fungi of the Dominant Grass *Stipa krylovii* From a Mongolian Steppe Grassland

**DOI:** 10.3389/fmicb.2019.02565

**Published:** 2019-11-12

**Authors:** Dániel G. Knapp, Ildikó Imrefi, Enkhtuul Boldpurev, Sándor Csíkos, Galiya Akhmetova, Péter János Berek-Nagy, Burenjargal Otgonsuren, Gábor M. Kovács

**Affiliations:** ^1^Department of Plant Anatomy, Institute of Biology, Eötvös Loránd University, Budapest, Hungary; ^2^Department of Ecology, Mongolian University of Life Sciences, Ulaanbaatar, Mongolia

**Keywords:** Asia, diversity, phylogeny, Pleosporales, root-associated fungi

## Abstract

In several terrestrial ecosystems such as grasslands, plants live together with various root-colonizing dark septate endophytes (DSEs), fungi that are relatively frequent colonizers of healthy belowground tissues of plants in these environments. They are important members of the plant microbiota and may have various effects on plant survival under different stress conditions; however, their general functions in relation to plants and the greater ecosystem remain elusive. Although an increasing number of studies has been published focusing on DSEs in Asian grasslands, our knowledge is limited. Especially in Mongolia, where the steppe region represents a significant area, information is not available on these root colonizers. In this study, we aimed to characterize DSEs of a common dominant gramineous plant species, *Stipa krylovii* in a semiarid grassland of Mongolia. Root samples were collected in a natural steppe and were processed for isolation of fungal endophytes. For molecular identification of the isolates, the internal transcribed spacer (ITS) region of the nrDNA was obtained for all the isolates investigated; furthermore, the partial translation elongation factor 1-α (TEF) gene and large subunit (LSU) and small subunit (SSU) of rDNA were also amplified and sequenced in case of representative isolates. *In vitro* tests were used to examine the rough symbiotic nature of the fungi, and root colonization was visualized. A majority of the 135 isolates examined in detail was found to belong to several orders of Ascomycota (110 isolates) and some to Basidiomycota (25 isolates). A significant number of the isolates represented presumably novel taxa, and dominant similarities of the lineages have been found with relatively frequent and known grass root endophytes of semiarid areas in other geographic regions. These endophytes included *Periconia macrospinosa*, *Microdochium bolley*, and *Darksidea*, the genus of which comprised one fourth of the isolates. We found numerous lineages, which have been detected not only from Asian steppe ecosystems, but also from prairies in North America and sandy grasslands in Europe. Therefore, our results strengthen the hypothesized worldwide presence of a common and dominant core group of a DSE community in arid and semiarid grasslands.

## Introduction

In grasslands, as in other terrestrial ecosystems, plants form symbioses with diverse fungal endophytes, which colonize the plant tissues without causing obvious symptoms during at least one part of their life cycle (Wilson, 1995; Saikkonen et al., 1998). Endophytic fungi are also present in healthy belowground tissues (Vandenkoornhuyse et al., 2002; Rodriguez et al., 2009), albeit knowledge of their general occurrence and their potential functions is lacking compared with what we know of mycorrhizal fungi. Apart from behaving as commensalistic symbionts, fungal endophytes also act as latent pathogens, latent saprotrophs, and mutualistic partners (Porras-Alfaro and Bayman, 2011; Yakti et al., 2019a). These form a group of root-colonizing endophytic fungi, generally called dark septate endophytes (DSEs), which refer to their mainly melanized and septate hyphae. These fungi dominate several biomes and climatic regions, including grasslands, yet their functions in relation to plants and the greater ecosystem are still elusive (Mandyam and Jumpponen, 2005; Sieber and Grünig, 2013). They might have an important role as saprobes because comparative genomics, for example, of DSE fungi revealed an expansion of carbohydrate active enzyme families (Knapp et al., 2018). Therefore, degrading complex carbohydrates such as dead plant tissues could be a key characteristic of the lifestyle of DSE fungi (Knapp et al., 2018). The study of enzymes and carbon source use of DSEs also revealed a diverse enzymatic capacity showing complementary distribution within DSEs of grass and non-grass hosts (Knapp and Kovács, 2016). The effect of DSE fungi on the performance of their host plants varies (Newsham, 2011; Mayerhofer et al., 2013; Mandyam and Jumpponen, 2015); in addition to influencing nutrient uptake (Yakti et al., 2019a, b), they could increase the drought stress resistance as well (Li et al., 2018).

In arid, semiarid, and temperate grasslands of North America and Europe, DSE communities and non-mycorrhizal root-associated fungi have been thoroughly studied, and these fungi are relatively frequent in these ecosystems (e.g., Kovács and Szigetvári, 2002; Mandyam and Jumpponen, 2005; Porras-Alfaro et al., 2008; Sánchez-Márquez et al., 2008; Knapp et al., 2012). The results suggest that there are core members of those communities common to disparate regions, not only in North America (Khidir et al., 2010) but also worldwide (Knapp et al., 2012). In the past few years, an increasing number of studies has been published focusing on fungal root endophytes of Asian grasslands, mainly in China (e.g., Su et al., 2010; Li et al., 2015, 2018; Xie et al., 2017). However, information about DSEs from other sites and countries in the eastern part of the Steppe belt, including Mongolia, where the steppe represents a significant part of the area (Ulziikhutag, 1989), is not available.

In Mongolia, in addition to alpine tundra, mountain taiga and deserts, three types of grass-dominated ecosystems can be found: mountain forest-steppe, steppe, and desert steppe. These are formed because of climate shifts from humid to arid conditions, and grazing may also affect the water cycle of the grassland ecosystem (Ulziikhutag, 1989). Areas of Mongolia are classified as arid and semi-arid regions (Begzsuren et al., 2004) and about 70–80% of the total land area in Mongolia is made up of grasslands comprising the three steppe types (Ulziikhutag, 1989; Liu et al., 2013), which are freely grazed by livestock year round (Fernandez-Gimenez and Allen-Diaz, 1999). Similar to the grassland ecosystems in China, a couple of grass species, including *Stipa krylovii* and *Stipa grandis*, dominate the landscape at certain areas in Mongolia (Zhao et al., 2006; Kang et al., 2007; Tuvshintogtokh, 2014). Other dominant gramineous species of the steppes are *Cleistogenes squarrosa*, *Leymus chinensis*, *Agropyron cristatum*, *Caragana microphylla*, and *Caragana stenophylla* (Tuvshintogtokh, 2014).

*Stipa krylovii* is an important perennial tussock grass in the Mongolian steppe ecosystem and its communities represent a major grassland type in the moderate temperate zone of Central Asia (Zhao et al., 2006). This grass is a primary forage in some Mongolian steppe areas for grazing mammals (e.g., Retzer, 2007) along with *A. cristatum*, which is widely used in the restoration of the Mongolian prairie (Otgonsuren and Lee, 2010). *S. krylovii* has been widely studied in several works owing to its outcrossing mating system, and ecological and economic importance (e.g., Yuan et al., 2005; Wang et al., 2006; Zhan et al., 2007; Li et al., 2014). Although studies focusing on this grass were started in the 1950s, with documentation of its distribution, growth, physiology, life history, and the response to grazing (Zhao et al., 2006), its root-colonizing endophytic communities remained unknown. Besides understanding the genetic diversity of population, onto which several works addressed questions (e.g., Wang et al., 2006; Zhao et al., 2006), studies on the relationship of this grass species with its microbiota, including fungal root endophytes, should be highly important owing to the hypothesized major impact of DSEs on their host plants in arid conditions (e.g., Li et al., 2018).

Card et al. (2014), in their study, investigated *Epichloë*/*Neotyphodium* endophytes colonizing shoots of grasses in several countries, including Mongolia; however, to date, we are not aware of any studies focusing on root endophytes of Mongolian grasslands. In the present study, we introduce the first information on DSEs of a dominant and widespread grass species from Mongolia. Similar to China (Zhao et al., 2005), desertification is an important issue in Mongolia, where 90% of the land is fragile dry land under increasing threat from this kind of land degradation (Chen et al., 2007). Therefore, information on the DSE community, which can affect plant survival and production, and its putative similarity to other grasslands, like those in European and North American prairies, may help applied approaches in farming or conservation.

In this study, we examined the root-colonizing fungal endophytes of a common and dominant gramineous plant species of semiarid grasslands in Mongolia. Our aims were to isolate fungal endophytes from the roots of *S. krylovii* from a natural Mongolian Steppe, carry out their molecular phylogenetic identification and to test their endophytic lifestyle in an artificial *in vitro* resynthesis experiment.

## Materials and Methods

### Sampling

The root samples were taken from *S. krylovii* from a grassland ecosystem near Kherlenbayan-Ulaan (KBU, Mongolia), where this is the dominant gramineous species. Samples were collected in a natural steppe zone located in the Nalaikh district, ∼38.6 km from the capital city of Ulaanbaatar, Mongolia. The region has semiarid characteristics with warm summers (Bereneva, 1992), the mean annual precipitation is 235 mm, and the mean temperature in January and July are −22.5°C and 17°C, respectively (averages for 2009–2018 at Nalaikh; National Agency for Meteorology and Environmental Monitoring, Mongolia). Sampling of *S. krylovii* roots was carried out on the 8th of October in 2016. The elevation of the collection site (N 47.729611, E 107.225104) is 1400–1450 m. Root samples were collected from 20 tussocks of *S. krylovii* with ∼1 m distance from one another along a transect. A bunch of root pieces and a small amount of moist soil were collected, put into plastic bags and kept in a cool condition. Samples, containing approximately 5–50 pieces of root per tussock, were cleaned from soil, folded into moist paper towels, and the roots were processed within 8 days.

### Isolation of Root Endophytes

Roots of *S. krylovii* were surface sterilized according to Knapp et al. (2012), and each sample was sliced into 2- to 3-cm segments and soaked in 30% H_2_O_2_ for 2 min, then in 70% alcohol for 1 min, and washed in sterile tap water two times for 2–3 min each time. After surface sterilization, each root segment was cut into four pieces by a scalper and placed onto modified Melin–Norkrans (MMN) media (Marx, 1969). After 5–7 days, we observed the growing hyphae from the roots and the pure mycelia were transferred to new agar plates.

### *In vitro* Tests and Microscopy

**FIGURE 1:**
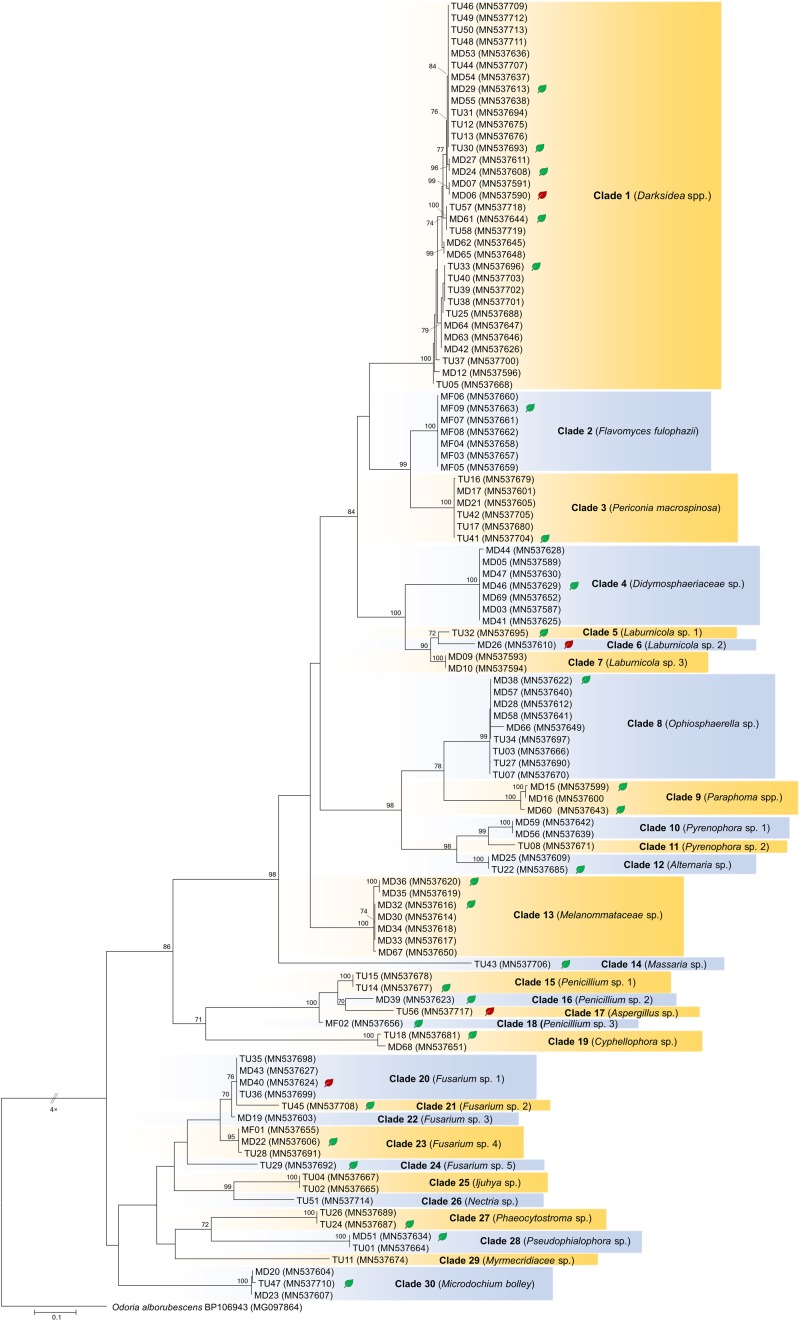
Maximum likelihood (RAxML) phylogenetic tree of ITS sequences of isolates belonging to Ascomycota. ML bootstrap support values (≥70) are shown at the branches. The basidiomycete *Odoria alborubescens* BP106943 served as outgroup. After the isolate names, GenBank accession numbers are shown in brackets. Leaves indicate the representative isolates tested by inoculation of leek; isolates with negative effect are labeled with red, and green leaves indicate no visible symptoms caused by the isolate. The scale bar indicates 0.05 expected changes per site per branch.

**FIGURE 2:**
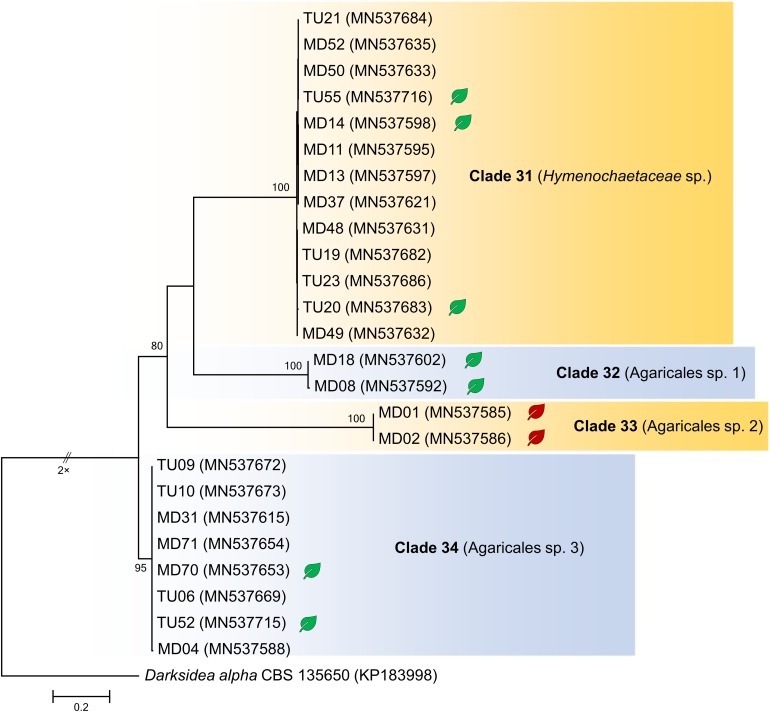
Maximum likelihood (RAxML) phylogenetic tree of ITS sequences of isolates belonging to Basidiomycota. ML bootstrap support values (≥70) are shown at the branches. The ascomycete *Darksidea alpha* CBS 135650 served as outgroup. After the isolate names, GenBank accession numbers are shown in brackets. Leaves indicate the representative isolates tested by inoculation of leek; isolates with negative effect are labeled with red, and green leaves indicate no visible symptoms caused by the isolate. The scale bar indicates 0.05 expected changes per site per branch.

*In vitro* tests were performed with representatives of each clade obtained by the analyses of internal transcribed spacer (ITS) sequences of the fungal isolates (Figures 1, 2) using leek (*Allium porrum*), a generally used host plant in DSE resynthesis experiments (see Mandyam and Jumpponen, 2008; Knapp et al., 2012) to test the basic symbiotic nature of the fungi. Five replicates for each fungal isolate and five control plants were incubated in each series according to Knapp et al. (2012). The fungus and isolates of its clade were considered a root endophyte if it colonized the roots without symptoms.

Root samples from the field and *in vitro* experiments were studied microscopically. The cleared roots were stained using the fluorescence labeled lectin, WGA-AlexaFluor488 (Wheat Germ Agglutinin, Alexa Fluor 488 conjugate, Molecular Probes W11261, Thermo Fisher Scientific, Lithuania), a cell-wall-specific dye used for *in planta* visualization of fungal endophytes (e.g., Andrade-Linares et al., 2011). Root samples were examined using a light microscope with Nomarski (differential interference contrast, DIC) optics and a Nikon Eclipse 80i microscope equipped with a Spot 7.4 Slider camera (Diagnostic Instruments, Inc.), differential interference contrast (DIC), and a filter wheel with excitation and emission filters for visualization of Alexa Fluor 488 probe.

### DNA Extraction, Amplification, and Sequencing

Genomic DNA was extracted from fungal mycelia using a modified cetyl trimethylammonium bromide (CTAB) method (Murray and Thompson, 1980; Kovács et al., 2001) or the NucleoSpin Plant II DNA Isolation Kit (MACHEREY-NAGEL, Germany) following the manufacturer’s instructions. The nuclear rDNA ITS1-5.8S-ITS2 (ITS) region of all isolates was amplified and sequenced using the primer pair ITS1F (Gardes and Bruns, 1993) and ITS4 (White et al., 1990). For representative strains of distinct lineages, the partial 28S rDNA (LSU), partial 18S rDNA (SSU), and the partial translation elongation factor 1α (TEF) were amplified with the primers LR0R (Rehner and Samuels, 1994) and LR5 (Vilgalys and Hester, 1990), NS1 and NS4 (White et al., 1990), and EF1-983F and EF1-2218R (Rehner and Buckley, 2005), respectively. For polymerase chain reaction, DreamTaq polymerase (Thermo Fisher Scientific, Vilnius, Lithuania) was used, and sequencing of the amplicons was carried out with the amplification primers from LGC GmbH (Berlin, Germany). The sequences were compiled from electropherograms using the PREGAP4 and GAP4 tools of the Staden software package (Staden et al., 2000) and deposited in GenBank (ITS: MN537585–MN537719; LSU: MN515229–MN515287; SSU: MN515296–MN515302; TEF: MN535230–MN535285; see Supplementary Table S1). The sequences obtained were compared with sequences in public databases using BLASTn searches (Altschul et al., 1990).

### Phylogenetic Analyses

Different data sets were prepared for phylogenetic analyses including sequences of the isolates collected from *S. krylovii* roots studied here and similar sequences obtained from public databases (Supplementary Table S2). Alignments of the sequences were assembled using the E-INS-i method in MAFFT 7 (Katoh and Standley, 2013) and were checked and edited with MEGA6 (Tamura et al., 2013) and deposited in TreeBASE (study S25135). For the data sets, multilocus phylogenetic Bayesian inference (BI) analyses were performed with MrBayes 3.1.2 (Ronquist and Huelsenbeck, 2003) using the GTR + G nucleotide substitution model. Four Markov chains were run for 10,000,000 generations and sampled every 1000 generations with a burn-in value set at 6000 sampled trees. Maximum likelihood (ML) phylogenetic analyses were carried out with the raxmlGUI 1.3 (Silvestro and Michalak, 2012) implementation of RAxML (Stamatakis, 2014). The GTR + G nucleotide substitution model was used for the partitions with ML estimation of base frequencies, and a ML bootstrap analysis with 1000 replicates was used to test the support for the branches. The phylogenetic trees were visualized and edited using MEGA6 (Tamura et al., 2013).

## Results

During the isolation process, more than 1000 root sections were surface sterilized and laid onto media, and approximately 350 isolates were obtained from the 20 *S. krylovii* tussocks that originated from a Mongolian grassland. In most cases, isolates collected from the same root of one *S. krylovii* tussock showed identical colony morphology. Because endophytes from the same root with similar colony morphology were considered to represent the same taxa, 135 isolates were finally used in the subsequent molecular identification and analysis. Isolates were obtained from all the tussocks sampled, and each of the field collected roots showed frequent colonization by DSE fungi.

The collected isolates belonged to the Dikarya group and represented diverse orders of Ascomycota, Pezizomycotina (110 isolates), Basidiomycota, and Agaricomycotina (25 isolates). Based on ITS sequences, the isolates represented 34 clades introduced here as clades 1–34, from which 30 comprised ascomycetous and 4 comprised basidiomycetous fungi (Figures 1, 2). The most numerous clade, possessing almost one fourth of the collected fungi, consists of 33 isolates (clade 1), followed by clade 31 and clade 8 with 13 and 9 isolates, respectively (Figures 1, 2). The majority of the isolates (125) belonged to non-singleton clades while 10 clades contained only one isolate.

**FIGURE 3:**
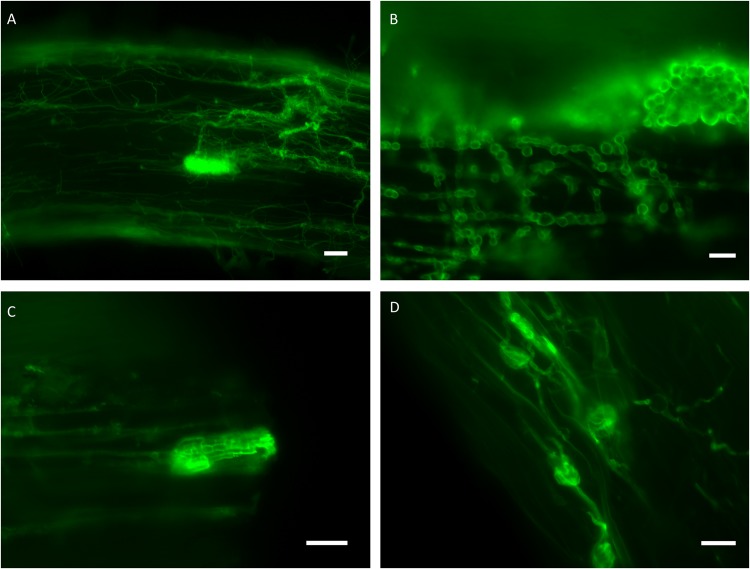
Representative isolates of different clades colonizing the roots of leek visualized by WGA-Alexa Fluor^®^ 488. **(A)** Intra- and extraradical hyphae and a microsclerotia formed by the isolate MD39 (clade 16). **(B)** Moniliform hyphae and an extraradical compacted structure of the isolate MF09 (clade 2). **(C)** Intraradical hyphae and a microsclerotia formed by the isolate MF09 (clade 2). **(D)** Intraradical hyphae and chlamydospore-like structures of the isolate TU56 (clade 17) within the dead root. Bars: 20 μm **(B**–**D)**, 50 μm **(A)**.

For the *in vitro* colonization tests, 39 representative isolates of the 34 clades were chosen (Figures 1, 2). The healthy *A. porrum* plants had generally well-developed root systems and five to six leaves after 6 weeks. All the fungi used for inoculation colonized the roots extra- and intraradically, showing typical structures, such as microsclerotia, chlamydospores, or intracellular septate hyphae (Figure 3). Altogether, only six isolates, representing five groups [clades 6, 17, 20, 33 and the MD06 from clade 1 (Figures 1, 2)], showed negative effects on the hosts; the plants inoculated with these isolates died within 2 weeks. In most cases, the leeks did not show considerable difference from the control plants.

**FIGURE 4:**
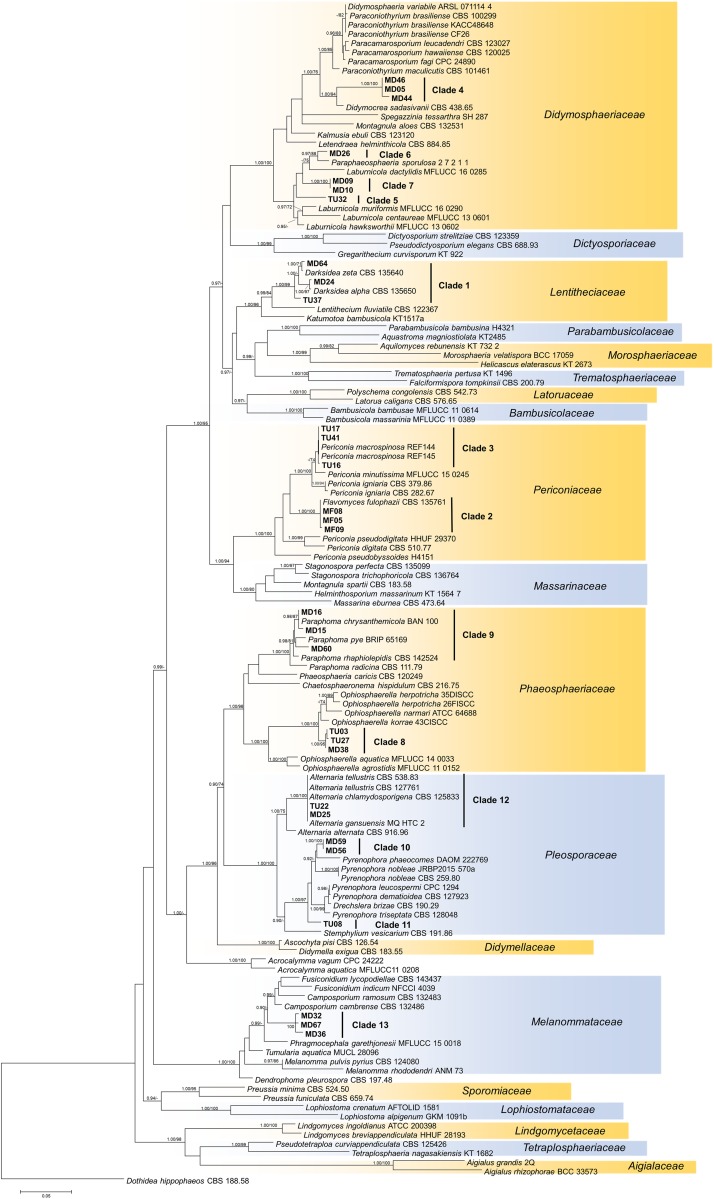
Maximum likelihood (RAxML) phylogenetic tree of representative sequences from Pleosporales based on the analysis of three loci (LSU, ITS, and TEF). Bayesian posterior probabilities (≥90) are shown before slashes; ML bootstrap support values (≥70) are shown after slashes. *Dothidea hippophaeos* CBS 188 58 served as outgroup. Clades based on the ITS analysis of the isolates are labeled. The names of isolates collected in this study are bolded. The scale bar indicates 0.05 expected changes per site per branch.

**FIGURE 5:**
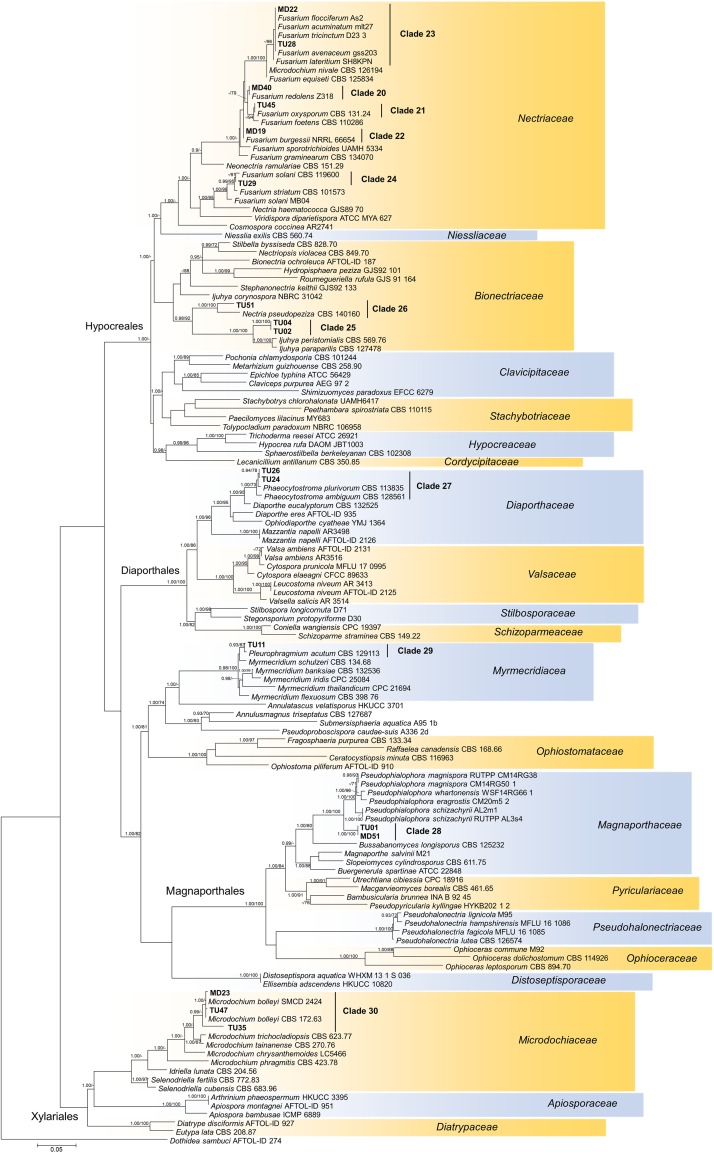
Maximum likelihood (RAxML) phylogenetic tree of representative sequences from Sordariomycetes based on the analysis of three loci (LSU, ITS, and TEF). Bayesian posterior probabilities (≥90) are shown before slashes; ML bootstrap support values (≥70) are shown after slashes. *Dothidea sambuci* AFTOL ID 274 served as outgroup. Clades based on the ITS analysis of the isolates are labeled. Isolates collected in this study are bolded. The scale bar indicates 0.05 expected changes per site per branch.

**FIGURE 6:**
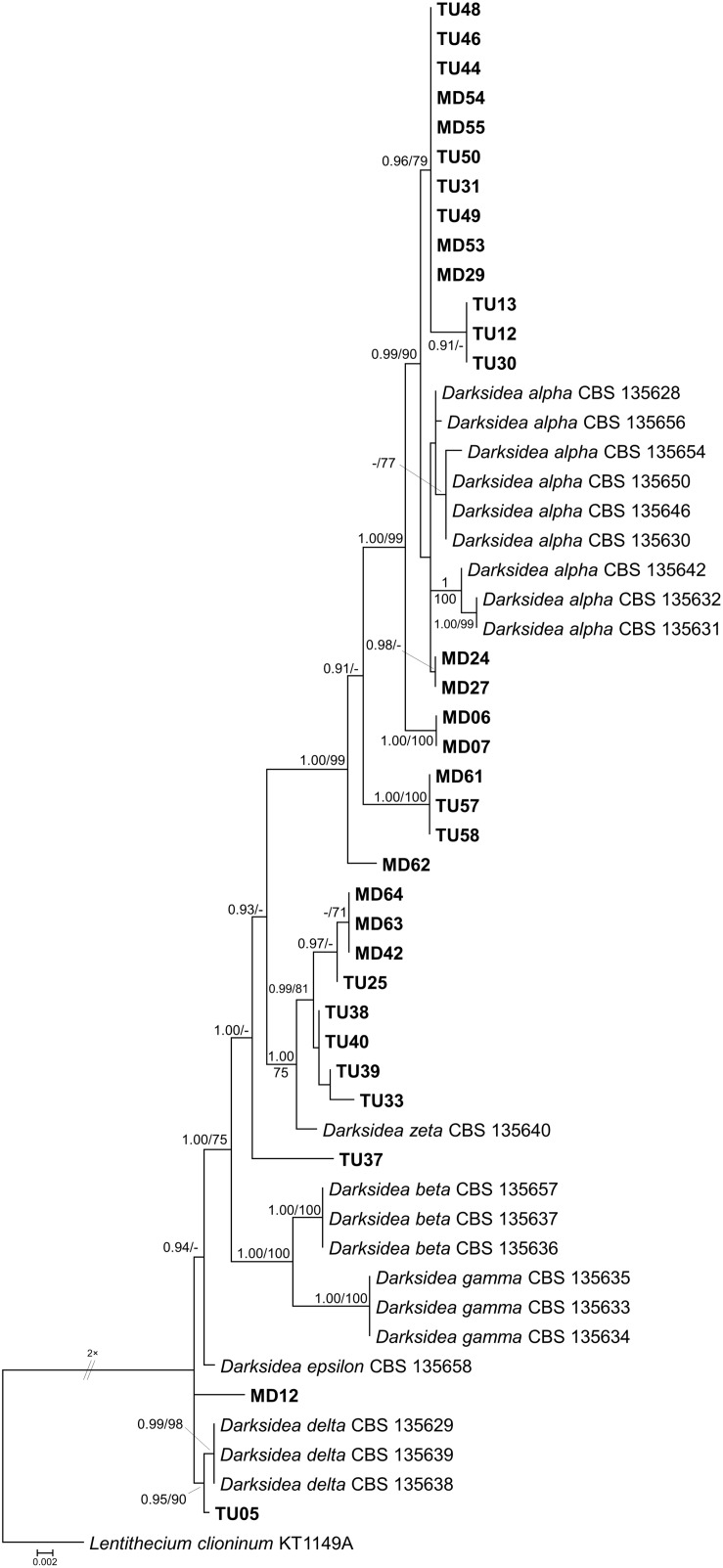
Maximum likelihood (RAxML) phylogenetic tree of *Darksidea* isolates and representative sequences from Sordariomycetes based on the analysis of three loci (LSU, ITS, and TEF). Bayesian posterior probabilities (≥90) are shown before slashes; ML bootstrap support values (≥70) are shown after slashes. *Lentithecium clioninum* KT1149A served as outgroup. The names of isolates collected in this study are bolded. The scale bar indicates 0.002 expected changes per site per branch.

The isolates showed diverse colony morphology, growing characteristics, color, and shape on agar plates. The colony of some isolates covered the whole 5-cm Petri dish, and other colonies remained only 1.0–1.5 cm in diameter. The presence of visible/coloring exudates was not common, and sporulation was observed only in case of a few *Aspergillus* and *Penicillium* isolates. Some isolates stained the media (e.g., isolates of clades 1 and 2). Based on molecular phylogenetic identification, the majority of the clades could be identified at species or genus level, whereas others could be identified only at higher taxonomic levels (Supplementary Table S3). Pleosporales (Dothydeomycetes) was the most represented order with 81 isolates and 13 clades (81/13), followed by Hymenochaetales (13/1) and Hypocreales (9/7). The blast analysis revealed similarities in the isolates with root endophytes from grasslands of other geographic regions; for instance, *Periconia macrospinosa* (clade 3), *Microdochium bolley* (clade 30), or the recently described genus, *Darksidea* (clade 1), from taxa that are relatively frequent and are known root endophytes of grasses of semiarid areas (Mandyam et al., 2010; Knapp et al., 2012, 2015; Jumpponen et al., 2017). Altogether, 20 lineages (clades 1, 2, 3, 4, 5, 9, 10, 11, 12, 13, 15, 19, 22, 25, 26, 27, 29, 30, 33, and 34) comprising more than two thirds of the isolates gathered (95) showed unambiguous ITS sequence similarities (at least 99%) with blast hits from different grassland ecosystems (Supplementary Table S3). Based on the blast analyses and the multilocus phylogenies, a significant number of the clades represents presumably novel taxa (Figures 4, 5, 6 and Supplementary Table S3).

Our isolates represented several families within Pleosporales (Figure 4). The largest group with the highest number of isolates was clade 1 representing *Darksidea* species within *Lentitheciaceae*. The isolates in clades 5–7 belong to the genus *Laburnicola* representing the *Didymosphaeriaceae* along with clade 4, a distant and novel lineage. Clades 2 and 3 represent *Flavomyces fulophazii* and *P. macrospinosa* in *Periconiaceae*, while *Phaeosphaeriaceae* and *Pleosporaceae* comprise clades 8 and 9, and clades 10–12, respectively. The three isolates of clade 13 represent another novel taxon in *Melanommataceae* (Figure 4).

Phylogenetic analysis of our representative isolates and related sequences in Sordariomycetes revealed numerous novel lineages (Figure 5). Clades 20–24 comprised possibly different *Fusarium* species in *Nectriaceae*, and isolates of clades 25 and 26 represented the family *Bionectriaceae*. Low numbers of endophytic isolates in clades 27–29 represented *Myrmecridiaceae*, *Diaporthaceae*, and *Magnaporthaceae*, respectively. The three isolates representing clade 30 showed similarities with *M. bolley* and belonged to *Microdochiaceae* (Figure 5).

As mentioned above, clade 1 encompassed a large number of isolates and represented the diverse genus, *Darksidea* (Figure 6). The phylogenetic analysis based on the ITS, TEF, and LSU regions of the isolates showed that most of the isolates represented *D. alpha* and grouped together with the well-described strains, including the ex-type strain CBS 135650. In addition to the *sensu stricto D. alpha* group, MD06 and MD07 represent a closely related lineage similarly to MD61, TU57, and TU58, and the solely branching MD62. These lineages may belong to *D. alpha* or represent novel taxa (Figure 6). Eight isolates represented a diverse lineage as a sister group of the monotypic species *D. zeta*. The isolate TU05 forms a clade with *D. delta* isolates, and two distinct isolates, TU37 and MD12, might represent novel species within *Darksidea* (Figure 6).

**FIGURE 7:**
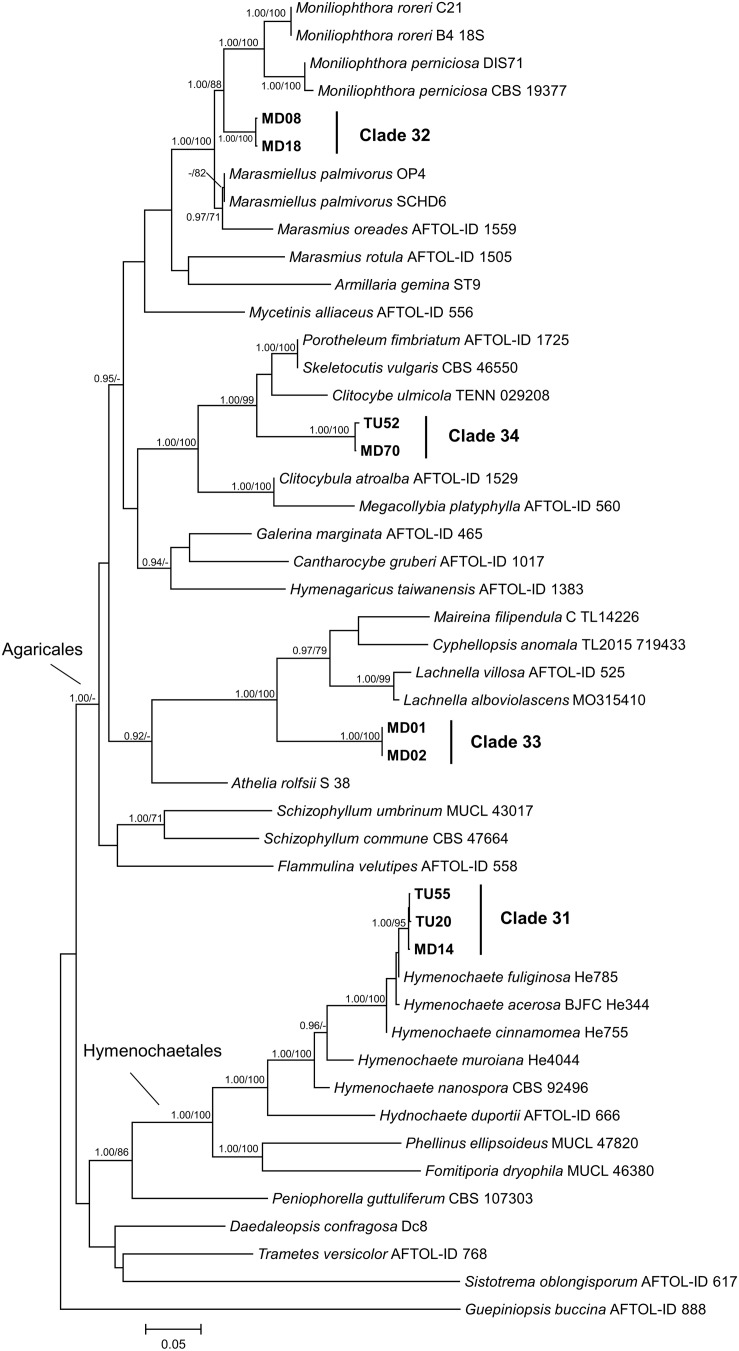
Maximum likelihood (RAxML) phylogenetic tree of representative basidiomycetous isolates from Mongolia and representative sequences of the most similar BLAST matches from GenBank. The analysis was carried out based on four loci (LSU, ITS, SSU, and TEF). Bayesian posterior probabilities (≥90) are shown before slashes; ML bootstrap support values (≥70) are shown after slashes. *Guepiniopsis buccina* AFTOL ID 888 served as outgroup. Clades based on the ITS analysis of the isolates are labeled. Isolates collected in this study are bolded. The scale bar indicates 0.05 expected changes per site per branch.

The multilocus phylogenetic analysis showed that, among related basidiomycetous sequences from GenBank, our isolates represented four lineages (Figure 7), which were already shown by the ITS phylogeny (Figure 2). Each lineage represented distinct lineages and potential new taxa within Agaricomycotina. Clades 32, 33, and 34 represented distant families in Agaricales, but their phylogenetic position is ambiguous. Clade 31, represented by three isolates, belonged to *Hymenochaetaceae* and formed a well-supported new lineage within *Hymenochaete*.

## Discussion

Dark septate endophytes are a widely distributed group of fungi, and, based on their dominance in belowground tissues of plants of grasslands worldwide (Jumpponen, 2001; Kovács and Szigetvári, 2002; Mandyam and Jumpponen, 2005; Herrera et al., 2010; Su et al., 2010; Fracchia et al., 2011; Knapp et al., 2012; Loro et al., 2012), these endophytes are hypothesized to be important functional members of fungal communities of harsh, nutrient-limited environments, such as arid and semiarid areas. The presence of the DSE hyphae and different structures in *S. krylovii* roots seems remarkable, similar to previous findings both in relatively close and distant grasslands. Hou et al. (2019), in their study, found extensive colonization in three plant species, including grasses investigated in semiarid continental sand lands of northern China. The abundant DSE colonization of gramineous plants has been reported from grassland ecosystems in various countries and continents (e.g., Kovács and Szigetvári, 2002; Mandyam and Jumpponen, 2008; Loro et al., 2012; Li et al., 2015; Xie et al., 2017).

Although knowledge of root endophytes of gramineous plants in Mongolian grasslands is poor, important findings have been published about the root-associated fungal communities in the so-called Inner Mongolian Steppe located in China (e.g., Su et al., 2010; Li et al., 2015; Xie et al., 2017). Su et al. (2010) investigated the endophytic fungi associated with *S. grandis* in the Inner Mongolia Steppe and, similar to our results, they found several lineages such as *Darksidea* species, *Fusarium redolens*, *M. bolley*, *P. macrospinosa*, and a basidiomycetous linage representing clade 34 in this study.

Because the isolates were collected from healthy, symptomless root sections, we consider here these fungi to be root endophytes according to the loose definition of endophytic fungi (see Wilson, 1995; Saikkonen et al., 1998). Based on the results of the *in vitro* tests we performed in this study with representative isolates and the non-host monocot leek, which was previously used for artificial tests for DSE fungi (Mandyam et al., 2010; Knapp et al., 2012), we found that representatives of most lineages showed, with few exceptions, no obvious negative effects on the leek. Six isolates caused visible symptoms and represented five clades belonging to novel basidiomycetous and *Laburnicola* lineages, and to the complex *Fusarium* and *Aspergillus* genera, none of which are typical or known DSEs. However, in many cases, *Fusarium* species live within plant tissues without causing visible symptoms to the host, and can be important members of the endophytic community of the root and shoot (Knapp et al., 2012; Pereira et al., 2018). They can even be beneficial to the plant (Redman and Rodriguez, 2010). The negative effect of the isolate MD06, which represents a distinct *Darksidea* lineage together with MD07 was unexpected. This is the first note on negative effect in case of *Darksidea* isolates, which have never been considered as pathogen using several isolates in the same experimental setup (Knapp et al., 2012) and other *in vitro* systems (e.g., Li et al., 2018).

Only some fungal taxa were represented by most of the isolates; thus, these groups could be categorized as dominant or at least common members of the root endophytic community of the area. Ten clades were represented by only one isolate. However, isolation frequency does not necessarily mean the level of abundance *in situ* and could be the result of the bias of the isolation technique or the number of samples taken. In this study, we did not address quantitative questions and abundance of certain lineages; nevertheless, the dominance of some clades, such as the *Darksidea* species complex (clade 1) or *Hymenochaete* sp. (clade 31), was obvious.

One fourth of the isolates belongs to several lineages of the recently described and worldwide distributed diverse genus, *Darksidea* (Knapp et al., 2015), which consists mainly of grass or grassland associated fungi that dominate roots in prairies and steppes of Europe and North America (Porras-Alfaro et al., 2008; Knapp et al., 2012, 2015), and also occur in grasses of coastal sand dunes and marine cliffs (Sánchez-Márquez et al., 2008; Pereira et al., 2018). Species of *Darksidea* are common and dominant members of the DSE communities in grassland ecosystems, and have been reported from several countries (see Knapp et al., 2015). The genus was also found in the eastern region of the Steppe belt in roots of the grass *S. grandis* in the semiarid steppe zone of the Inner Mongolian Plateau (Su et al., 2010). Li et al. (2018) reported on *Darksidea* isolates collected from a super-xerophytic shrub, *Gymnocarpos przewalskii* in Anxi Extra-Arid Desert National Nature Reserve and the Minqin Liangucheng National Nature Reserve, Gansu Province, northwest China. Hou et al. (2019) also isolated related fungi from roots of the clonal semishrub *Hedysarum leave* and the gramineous *Psammochloa villosa* in the Mu Us sandland, also in northwest China. This genus comprises six described species (Knapp et al., 2015); however, far more distinct lineages could be revealed within *Darksidea* based on the numerous related sequences deposited in public databases (e.g., Porras-Alfaro et al., 2008; Herrera et al., 2010; Glynou et al., 2016). *Darksidea* isolates are also diverse in colony morphology and highly vary, which is in contradiction with the similarity of their ITS or other DNA regions (Knapp et al., 2015). Here, we present further *Darksidea* isolates, representing several novel lineages within the genus, and within the most frequent and complex species *D. alpha*. Based on their presence in *S. krylovii* roots, *Darksidea* species might have a fundamental role in the life and annual growth cycle of the tussock. They might have a key role as a decomposer of dead roots owing to the generally expanded carbohydrate active enzymes of DSE fungi (Knapp et al., 2018). Based on our findings, we can support the conclusion of Knapp et al. (2015) that *Darksidea* is a common member of the core DSE community hypothesized to be shared by semiarid grassland areas worldwide.

Clade 3 represents another lineage of grass-associated DSEs, *P. macrospinosa*, a genomic analysis of which has been published recently by Knapp et al. (2018). This was the first comparative genomics of a DSE species of grasses. *P. macrospinosa* is distributed worldwide and common in grass-dominated ecosystems of the North American prairies and European grasslands (Mandyam et al., 2010, 2012; Knapp et al., 2012; Mandyam and Jumpponen, 2014; Jumpponen et al., 2017) and might have an important role at these areas owing to its frequency. That species, among others, also was isolated by Su et al. (2010), from roots of another *Stipa* species, *S. grandis*, in Inner Mongolia. Jumpponen et al. (2017) during a field survey of rhizobiomes, comparing communities of different grassland sites across the prairies of the United States, found that after *Gibberella*/*Fusarium* species (∼23%), *Periconia* was the second most abundant genus, comprising almost 15% of the isolates collected. This reinforced the belief that it is of major importance in these fields. In *Periconiaceae*, another lineage, the recently described *F. fulophazii* (clade 2) was found (Knapp et al., 2015), which is also a common grass endophyte in sandy grasslands of the Great Hungarian Plain; however, prior to this study, it was found only in Hungary.

A significant portion, more than one-fifth of the isolates, were basidiomycetes, representing four distinct lineages. This is quite notable compared with root-colonizing endophytes isolated from grasses in sites of the Eurasian steppe belt (e.g., Sánchez-Márquez et al., 2008; Su et al., 2010; Knapp et al., 2012), where the number of isolates belonging to any basidiomycetous taxa was negligible. This phenomenon may be the result of the diverse isolation techniques mentioned in the literature, in which the media used for isolation of the fungi growing from the root also differs.

Isolates of clades 5–7 grouped with *Laburnicola* species based on the analyses of ITS, LSU and SSU sequences, and seem to represent novel species within the genus, which consists mainly of saprobes on *Laburnum* debris (Wanasinghe et al., 2016).

Although clade 25 comprised fungi with 100% ITS similarity with an isolate (MK808464) from a grassland in the North American Great Plains, their further closest matches (less than 92% similarity) were the *Ijuhya* species. This genus comprises the newly described *I. vitellina* (Ashrafi et al., 2017), which destructively parasitizes eggs inside cysts of the nematode *Heterodera filipjevi*, similarly to another recently introduced taxon, *Polyphilus sieberi*, that also behaves as common root endophyte but colonizing truffle ascomata, too (Ashrafi et al., 2018).

More than half (60%) of the isolates collected from *S. krylovii* belong to Pleosporales, which is the largest order of Dothideomycetes (Zhang et al., 2012), and one of the most represented orders in root-associated communities of grassland ecosystems (Porras-Alfaro et al., 2008; Knapp et al., 2012; Jumpponen et al., 2017). It is worth noting the absence of the widely studied helotialean DSE, *Phialocephala fortinii* s.l.–*Acephala applanata* species complex (PAC), the common and abundant group of endophytes in temperate and boreal coniferous forested ecosystems, as well as other species in Helotiales (Dean et al., 2013; Sieber and Grünig, 2013; Vohník et al., 2013). Instead of the helotialean dominance in forest ecosystems, the pleosporalean fungi seem to be the common dominant root endophytes in grassland ecosystems along the Holarctic regions (Porras-Alfaro et al., 2008; Mandyam et al., 2010; Knapp et al., 2012, 2015; Jumpponen et al., 2017).

## Conclusion

In the present work, we investigated root-colonizing fungal endophytes of a common grass species of the steppes of Mongolia, which represent extended grasslands suffering from desertification and damage from anthropogenic activities (Liu et al., 2013). Here, we gained isolates from the roots of *S. krylovii* from the Mongolian grassland ecosystem, and carried out molecular identification of the isolated fungi. Although a majority of the isolates could be identified at the genus or species level, distinct lineages, probably representing novel taxa, are present among these endophytes. We have identified numerous fungi, which were detected in steppes not only from the Asian steppe ecosystems, but also from the prairies of North America and the sandy grasslands of Europe. Common and dominant lineages of grassland endophytes were also found in this study. Therefore, our results indicate the presence of common and dominant members of the DSE community of grasslands worldwide and strengthen our previous hypotheses on that core fungal community of those areas (Knapp et al., 2012).

## Data Availability Statement

All datasets generated for this study are included in the article/Supplementary Material.

## Author Contributions

DK and GK conceived the study. DK, EB, BO, and GK contributed to the sampling design and wrote the manuscript. All authors contributed to the data collection, critically reviewed and edited the manuscript, and approved its publication. DK, II, EB, SC, PB-N, and GA performed the experiments. DK, II, and GK performed the data interpretation.

## Conflict of Interest

The authors declare that the research was conducted in the absence of any commercial or financial relationships that could be construed as a potential conflict of interest.

## Supplementary Material

TABLE S1 GenBank accession numbers of the different sequences of different isolates gained in this study.

TABLE S2 GenBank accession numbers of sequences used for phylogenetic analyses.

TABLE S3 Closest BLAST matches for ITS sequence of representative isolates of DSE clades.
