# Supplementary material for: Root-Colonizing Endophytic Fungi of the Dominant Grass Stipa krylovii From a Mongolian Steppe Grassland
Source: Front Microbiol. 2019 Nov 12;10:2565. doi: 10.3389/fmicb.2019.02565 (PMC6861457; doi:10.3389/fmicb.2019.02565)
Supplement: TABLE S3 — Closest BLAST matches for ITS sequence of representative isolates of DSE clades. [file Table_3.DOCX]

| SUPPLEMENTARY TABLE 3 \| Closest BLAST matches for ITS sequence of representative isolates of DSE clades | | | | |
| --- | --- | --- | --- | --- |
| Clade | **Isolate** | **Best blast matches** | **Similarity** | **Accession No.** |
| Clade 1 | MD24 | *Darksidea* *alpha* strain CBS | 98.67% | KP183968.1 |
|  |  | *Darksidea* sp. isolate DS319 | 99.82% | MK808689.1 |
|  |  | *Periconia* sp. isolate DS1181 | 99.64% | MK808173.1 |
|  |  | *Darksidea alpha* isolate REF139 | 99.64% | KX058043.1 |
|  |  | Pleosporales sp. KRi72H | 99.46% | GQ923982.1 |
|  |  | Root-associated fungal sp. MK-124 | 98.20% | EU144366.1 |
|  | MD64 | *Darksidea* *delta* CBS | 97.65% | NR_137075.1 |
|  |  | *Darksidea* *zeta* CBS | 98.85% | NR_137958.1 |
|  |  | *Darksidea* sp. isolate DS361 | 99.26% | MK808729.1 |
|  |  | *Pleosporales* sp. strain P2503 | 97.68% | KT269732.1 |
|  |  | Pleosporales sp. KRi72H | 97.13% | GQ923982.1 |
|  | TU37 | Pleosporales sp. S0i83H | 95.78% | GQ923984.1 |
|  |  | *Darksidea* *zeta* CBS 135640 | 98.14% | NR_137958.1 |
|  |  | *Darksidea* *zeta* isolate REF131 | 97.67% | JN859351.1 |
|  |  | Pleosporales sp. S0i83H | 97.49% | GQ923954.1 |
|  |  | *Darksidea* sp. isolate DS1424 | 97.25% | MK808358.1 |
| Clade 2 | MF05 | *Flavomyces* *fulophazii* CBS 135761 | 100% | NR_137960.1 |
|  |  | Hypocreaceae sp. 1 UFMGCB 6344 | 94.41% | KJ471485.1 |
| Clade 3 | TU42 | Pleosporales sp. O5i87H | 100% | GQ923975.1 |
|  |  | Periconia sp. isolate RM38 | 99.81% | MG664770.1 |
|  |  | *Periconia* *macrospinosa* strain SMCD 2423 | 99.81% | JQ658341.1 |
|  |  | *Periconia* *macrospinosa* strain KS00113 | 99.81% | FJ536208.1 |
|  |  | *Periconia* *macrospinosa* | 99.81% | FN393421.1 |
| Clade 4 | MD05 | *Paraconiothyrium* sp. ATCC MYA-4697 | 95.02% | HQ999974.1 |
|  |  | *Paraconiothyrium* sp. isolate DS1329 | 99.86% | MK808285.1 |
|  |  | *Paraconiothyrium* sp. strain JMB01_2B | 95.02% | MH268019.1 |
|  |  | Foliar endophyte of *Picea* *glauca* sp. | 95.19% | AY566890.1 |
| Clade 5 | TU32 | Pleosporales sp. REF141 | 99.25% | JN859361.1 |
|  |  | Pleosporales sp. isolate DS1410 | 99.20% | MK808350.1 |
|  |  | *Laburnicola* *hawksworthii* MFLUCC 13-0602 | 94.72% | NR_154127.1 |
|  |  | Pleosporales sp. CBS 382.69 | 93.26% | KY940765.1 |
| Clade 6 | MD26 | Fungal sp. S17 | 98.07% | KF887135.1 |
|  |  | Didymosphaeriaceae sp. strain FO8 | 96.86% | MF186878.1 |
|  |  | Pleosporales sp. strain CSR3_6 | 95.79% | MK460849.1 |
|  |  | Pleosporales sp. strain JP-Root-31 | 95.77% | MG833814.1 |
|  |  | Pleosporales sp. CBS 128811 | 96.79% | KY940807.1 |
| Clade 7 | MD10 | Pleosporales sp. isolate HW3PH | 95.57% | KU612401.1 |
|  |  | Pleosporales sp. XS55m2 | 98.29% | KJ188732.1 |
|  |  | Pleosporales sp. REF141 | 93.55% | JN859361.1 |
|  |  | Pleosporales sp. isolate DS1410 | 91.86% | MK808350.1 |
|  |  | *Laburnicola* *centaureae* MFLUCC 13-0601 | 91.91% | NR_154131.1 |

| Clade 8 | MD38 | *Ophiosphaerella* sp. strain P2165 | 98.50% | KT269428.1 |
| --- | --- | --- | --- | --- |
|  |  | *Ophiosphaerella* *korrae* isolate 43CISCC | 97.77% | KC841035.1 |
|  |  | *Ophiosphaerella* *herpotricha* isolate KS28 | 97.57% | KP690992.1 |
|  |  | *Ophiosphaerella* *narmari* strain ATCC 64688 | 97.01% | KC848510.1 |
|  |  | *Ophiosphaerella* *korrae* isolate TX_1_4 | 97.02% | KC841079.1 |
| Clade 9 | MD15 | *Paraphoma* *chrysanthemicola* strain CBS | 99.41% | KF251165.1 |
|  |  | *Paraphoma* sp. strain P1828 | 99.42% | KT269100.1 |
|  |  | *Paraphoma* *chrysanthemicola* strain BAN-100 | 99.42% | JN123358.1 |
|  |  | *Paraphoma* *chrysanthemicola* isolate NG_H17 | 99.42% | HQ115688.1 |
|  |  | *Paraphoma* *chrysanthemicola* isolate P6256 | 99.42% | MH063746.1 |
|  | MD60 | Phaeosphaeriaceae sp. strain F0741 | 98.50% | KU747667.1 |
|  |  | *Paraphoma* sp. PHY-39 | 98.50% | JX401946.1 |
|  |  | *Paraphoma* sp. strain P1056 | 97.93% | KT268375.1 |
|  |  | *Paraphoma* *rhaphiolepidis* strain CBS | 97.74% | KY979758.1 |
| Clade 10 | MD59 | *Drechslera* sp. bc_besc_80d | 99.67% | KF428285.1 |
|  |  | *Drechslera* sp. BAFC 3419 | 99.50% | FJ868975.1 |
|  |  | *Drechslera* *nobleae* strain CBS 259.80 | 91.71% | AY004792.1 |
| Clade 11 | TU08 | *Pyrenophora* *nobleae* isolate CK8 | 99.35% | MH474481.1 |
|  |  | *Bipolaris* sp. HSAUP074449 | 100% | GQ184732.1 |
|  |  | *Pyrenophora* *dematioidea* strain CBS 127923 | 93.92% | MH864751.1 |
|  |  | *Pyrenophora* *fugax* isolate JRBP2015.858 | 97.55% | MH399509.1 |
| Clade 12 | TU22 | *Embellisia* sp. isolate REF146 | 100% | JN859366.1 |
|  |  | *Alternaria* *chlamydosporigena* strain CBS 125833 | 100% | MH863800.1 |
|  |  | *Alternaria* *chlamydosporigena* isolate CK1397 | 100% | MH474032.1 |
|  |  | *Alternaria* sp. strain P1857 | 100% | KT269128.1 |
|  |  | *Alternaria* *chlamydosporigena* strain MQ-PPC-2 | 99.82% | KY420923.1 |
|  |  | *Alternaria* *chlamydosporigena* isolate LGM28 | 99.82% | MF036005.1 |
| Clade 13 | MD32 | Uncultured fungus clone OTU124 | 99.58% | MF971176.1 |
|  |  | Uncultured ascomycete clone Cn5 | 98.94% | EU520605.1 |
|  |  | *Camposporium* *cambrense* strain CBS 132486 | 93.70% | MH866029.1 |
|  |  | *Fusiconidium* *lycopodiellae* culture CBS:143437 | 93.75% | MH107892.1 |
|  |  | *Camposporium* *cambrense* strain FMR_12069 | 93.19% | KY853428.1 |
|  | MD67 | *Camposporium* *cambrense* strain CBS 132486 | 92.94% | MH866029.1 |
|  |  | *Camposporium* *cambrense* strain FMR_12069 | 92.45% | KY853428.1 |
| Clade 14 | TU43 | *Massaria aucupariae* voucher WU 30512 | 85.31% (2e-93) | HQ599384.1 |
|  |  | *Massaria aucupariae* voucher WU 30513 | 85.31% (2e-93) | HQ599383.1 |
|  |  | *Massaria ariae* CBS 125589 | 86.01% (9e-92) | NR_137061.1 |
| Clade 15 | TU14 | *Penicillium* sp. strain HSrS2-4.1-MR | 100% | MK793753.1 |
|  |  | *Penicillium* *pulvillorum* strain CBS 132165 | 100% | MH865967.1 |
|  |  | *Penicillium* sp. H17 | 100% | GU566281.1 |
|  |  | *Penicillium* *ochrochloron* strain P9_D1_18 | 100% | JF311909.1 |
|  |  | *Penicillium* sp. isolate DS707 | 99.82% | MK808877.1 |
| Clade 16 | MD39 | *Penicillium* *pasqualense* CBS 126330 | 100% | NR_121513.1 |
|  |  | *Penicillium* *pasqualense* strain CV2387 | 100% | JX140864.1 |
|  |  | *Penicillium* *pasqualense* strain CBS 126330 | 100% | JN617676.1 |
|  |  | *Penicillium* *pasqualense* strain CBS 126329 | 100% | MH863905.1 |
|  |  | *Penicillium* *vancouverense* CBS 126323 | 99.82% | NR_121512.1 |

| Clade 17 | TU56 | *Aspergillus* *niger* isolate Z4 | 99.82% | MH892847.1 |
| --- | --- | --- | --- | --- |
|  |  | *Aspergillus* *niger* strain FC7347 | 99.82% | MK693453.1 |
|  |  | *Aspergillus* *tubingensis* strain CBS 559.65 | 99.82% | MH858714.1 |
|  |  | *Aspergillus* *niger* strain CBS 117.36 | 99.82% | MH855726.1 |
| Clade 18 | MF02 | *Penicillium* *egyptiacum* strain CBS 457.72 | 100% | MH860529.1 |
|  |  | *Penicillium* *molle* CBS 456.72 | 100% | NR_138261.1 |
|  |  | *Penicillium* *kewense* strain CBS 344.61 | 100% | MH858076.1 |
|  |  | *Penicillium* *egyptiacum* genes | 99.33% | AB479319.1 |
| Clade 19 | TU18 | *Cyphellophora* sp. strain P1380 | 100% | KT268675.1 |
|  |  | Uncultured fungus clone OTU419 | 100% | MF971416.1 |
|  |  | *Cyphellophora* *europaea* strain CBS 101466 | 92.52% | EU514698.1 |
|  |  | *Cyphellophora* *europaea* IFM 64788 | 92.91% | LC413748.1 |
|  |  | *Phialophora* *europaea* | 92.61% | EF540756.1 |
| Clade 20 | TU35 | *Fusarium* *redolens* isolate YS16 | 100% | MH660908.1 |
|  |  | *Fusarium* *redolens* isolate Opheim-valley-MT | 100% | MK729587.1 |
|  |  | *Fusarium* *redolens* isolate Z318 | 100% | KP264660.1 |
| Clade 21 | TU45 | *Fusarium* *oxysporum* strain CBS 130301 | 100% | MH865885.1 |
|  |  | *Fusarium* *oxysporum* strain JFP9 | 100% | MK849925.1 |
|  |  | *Fusarium* *oxysporum* isolate PKP2 | 100% | MK590412.1 |
|  |  | *Fusarium* *oxysporum* strain MF22471 | 100% | MH911411.1 |
|  |  | *Fusarium* *oxysporum* isolate G303 | 100% | MH681160.1 |
| Clade 22 | MD19 | *Fusarium* sp. isolate DS989 | 100% | MK809067.1 |
|  |  | *Fusarium* sp. strain P2637 | 100% | KT269847.1 |
|  |  | *Fusarium* *redolens* isolate C03 | 99.43% | KY910892.1 |
|  |  | *Fusarium* *redolens* | 99.43% | HQ443207.1 |
| Clade 23 | MD22 | *Fusarium* *avenaceum* strain CBS 128538 | 100% | MH864972.1 |
|  |  | *Fusarium* *avenaceum* isolate Wolf Point7-MT | 100% | MK729617.1 |
|  |  | *Fusarium* *tricinctum* strain SDSF33 | 100% | MG840761.1 |
|  |  | *TEF sequence: *Fusarium* sp. strain P1388 | 100% | KX361599.1 |
|  |  | *TEF sequence: *Fusarium avenaceum* strain KR2013 | 98.04% | KM189442.1 |
| Clade 24 | TU29 | *Fusarium* *proliferatum* strain 144 | 99.81% | MK828121.1 |
|  |  | *Fusarium* *solani* strain NWFVA1696 | 99.81% | KU712219.1 |
|  |  | *Fusarium* *solani* strain zy39 | 99.81% | KP992939.1 |
|  |  | *Fusarium* *solani* strain CBS 127118 | 99.63% | MH864425.1 |
|  |  | *Fusarium* *solani* isolate BC-2(Fs) | 99.63% | MH782046.1 |
|  |  | *TEF sequence: *Fusarium solani* strain MB10A | 98.95% | LT615305.1 |
|  |  | *TEF sequence: *Fusarium euwallaceae* NRRL 62626 | 98.49% | KU171722.1 |
| Clade 25 | TU02 | Hypocreales sp. isolate DS1590 | 100% | MK808464.1 |
|  |  | *Ijuhya* *peristomialis* strain CBS 569.76 | 91.43% | KY607544.1 |
|  |  | *Ijuhya* *paraparilis* strain W8063/HMAS 183506 | 91.12% | FJ969801.1 |
|  |  | *Ijuhya* *paraparilis* strain CBS 127478 | 90.67% | MH864599.1 |
| Clade 26 | TU51 | *Scytalidium* sp. strain 2.1a | 100% | KX058044.1 |
|  |  | *Scytalidium* sp. YG-2010a | 99.64% | HQ213805.1 |
|  |  | *Scytalidium* *circinatum* isolate P6302 | 99.08% | MH063792.1 |
|  |  | *Scytalidium* *circinatum* strain MR8-1 | 98.86% | KT220670.1 |
|  |  | *Nectria* *pseudopeziza* strain CBS 126104 | 91.86% | MH864018.1 |

| Clade 27 | TU24 | *Phaeocytostroma* sp. isolate DS680 | 100% | MK808865.1 |
| --- | --- | --- | --- | --- |
|  |  | *Phaeocytostroma* *plurivorum* strain CBS 113835 | 99.08% | MH862945.1 |
|  |  | *Phaeocytostroma* *plurivorum* | 99.08% | FR748046.1 |
|  |  | *Aplosporella* *bakeriana* strain CBS 176.65 | 98.71% | MH858533.1 |
|  |  | *Phaeocytostroma* sp. MN_ROUGO3 | 98.89% | KR230075.1 |
| Clade 28 | TU01 | *Pseudophialophora* sp. isolate DS115 | 93.26% | MK808146.1 |
|  |  | *Pseudophialophora* *magnispora* RUTPP CM14RG38 | 93.32% | NR_158900.1 |
|  |  | *Pseudophialophora* tarda RUTPP WSF14SW13 | 93.32% | NR_158902.1 |
| Clade 29 | TU11 | *Myrmecridium* sp. TMS-2011 voucher SC17d100p18-10 | 100% | HQ631062.1 |
|  |  | *Myrmecridium* schulzeri strain CBS 642.76 | 99.82% | EU041777.1 |
|  |  | *Pleurophragmium* *acutum* strain CBS 129113 | 99.82% | MH865210.1 |
|  |  | *Myrmecridium* schulzeri isolate DS503 | 100% | MK808788.1 |
|  |  | *Myrmecridium* *schulzeri* strain Dzf10 | 99.62% | EU543253.1 |
| Clade 30 | TU47 | *Microdochium* *bolleyi* strain SMCD 2424 | 99.43% | JQ658340.1 |
|  |  | *Microdochium* *bolleyi* isolate C10 | 99.43% | HQ703412.1 |
|  |  | *Microdochium* *bolleyi* strain Q13-6 | 99.43% | KY365586.1 |
|  |  | *Microdochium* *bolleyi* isolate 20_45D | 99.43% | KC989068.1 |
|  |  | *Microdochium* *bolleyi* strain M29 | 99.24% | MH319854.1 |
| Clade 31 | TU55 | *Hymenochaete* sp. voucher ARAN-Fungi 7079 | 97.71% | MF990738.1 |
|  |  | *Hymenochaete* *cinnamomea* voucher He 2074 | 96.39% | KU975460.1 |
|  |  | *Hymenochaete* *fuliginosa* strain He785 | 98.19% | JQ279545.1 |
|  |  | *Hymenochaete* *nanospora* CBS 924.96 | 88.24% | MH862622.1 |
| Clade 32 | MD08 | *Marasmiellus* sp. JZBHM006 | 95.06% | KJ545432.1 |
|  |  | *Marasmiellus* sp. UOC MINNP MK06 | 94.89% | KR867662.1 |
|  |  | *Marasmius* *palmivorus* voucher AKD 112/2015 | 91.69% | MG251431.1 |
|  |  | *Moniliophthora* *perniciosa* strain CBS 193.77 | 87.64% | MH861049.1 |
| Clade 33 | MD01 | Uncultured fungus isolate KTRF57 | 99.67% (1e-153) | MG432984.1 |
|  |  | *Maireina filipendula* C TL14226 | 80.60% (3e-95) | NR_160459.1 |
|  |  | *Niaceae* sp. MD-2017a voucher TL2015-724890 | 80.60% (3e-95) | KX772746.1 |
|  |  | *Lachnella alboviolascens* strain MO315410 | 80.30% (2e-92) | MH558281.1 |
| Clade 34 | MD70 | *Tricholomataceae* sp. isolate DS68 | 98.05% | MK808864.1 |
|  |  | *Tricholomataceae* sp. 29 YS-2010 | 100% | HM007087.1 |
| *TEF sequence were used for BLAST search | | | | |
